# Proteomic and metabolomic analyses illustrate the mechanisms of expression of the *O^6^‐methylguanine‐DNA methyltransferase* gene in glioblastoma

**DOI:** 10.1111/cns.14415

**Published:** 2023-08-28

**Authors:** Xi Chen, Jinli Sun, Yukui Li, Weichao Jiang, Zhangyu Li, Jianyao Mao, Liwei Zhou, Sifang Chen, Guowei Tan

**Affiliations:** ^1^ Department of Neurosurgery The First Affiliated Hospital of Xiamen University Xiamen China; ^2^ Department of Reproduction The First Affiliated Hospital of Xiamen University Xiamen China

**Keywords:** DNA repair, glioblastoma, metabolomic, O^6^‐methylguanine‐DNA methyltransferase, proteomic

## Abstract

**Aim:**

Glioblastoma (GBM) has been reported to be the most common high‐grade primary malignant brain tumor in clinical practice and has a poor prognosis. O^6^‐methylguanine‐DNA methyltransferase (MGMT) promoter methylation has been related to prolonged overall survival (OS) in GBM patients after temozolomide treatment.

**Methods:**

Proteomics and metabolomics were combined to explore the dysregulated metabolites and possible protein expression alterations in white matter (control group), MGMT promoter unmethylated GBM (GBM group) or MGMT promoter methylation positive GBM (MGMT group).

**Results:**

In total, 2745 upregulated and 969 downregulated proteins were identified in the GBM group compared to the control group, and 131 upregulated and 299 downregulated proteins were identified in the MGMT group compared to the GBM group. Furthermore, 131 upregulated and 299 downregulated metabolites were identified in the GBM group compared to the control group, and 187 upregulated and 147 downregulated metabolites were identified in the MGMT group compared to the GBM group. The results showed that 94 upregulated and 19 downregulated proteins and 20 upregulated and 16 downregulated metabolites in the MGMT group were associated with DNA repair. KEGG pathway enrichment analysis illustrated that the dysregulated proteins and metabolites were involved in multiple metabolic pathways, including the synthesis and degradation of ketone bodies, amino sugar and nucleotide sugar metabolism. Moreover, integrated metabolomics and proteomics analysis was performed, and six key proteins were identified in the MGMT group and GBM group. Three key pathways were recognized as potential biomarkers for recognizing MGMT promoter unmethylated GBM and MGMT promoter methylation positive GBM from GBM patient samples, with areas under the curve of 0.7895, 0.7326 and 0.7026, respectively.

**Conclusion:**

This study provides novel mechanisms to understand methylation in GBM and identifies some biomarkers for the prognosis of two different GBM types, MGMT promoter unmethylated or methylated GBM, by using metabolomics and proteomics analyses.

## Graphical abstract

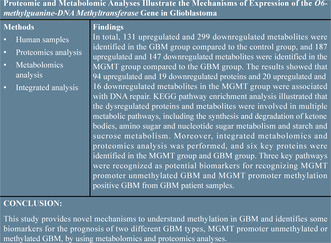

## 1 INTRODUCTION

Glioblastoma (GBM) has been reported to be the most widely occurring malignant primary brain tumor and causes high morbidity and mortality in clinical practice.^1^ According to the WHO Classification, GBM is the most aggressive diffuse glioma after astrocytic lineage and classes to grade IV.^2^ In the United States, the overall age‐adjusted incidence of GBM is 3.22/100,000 persons, and this incidence varies worldwide.^1^, ^3^ GBM's 5‐year survival rate of 6.8% is extremely low compared to all other tumor types.^1^ Moreover, the rising incidence of GBM is closely related to increasing age, achieving a peak incidence of 15.29/100,000 persons from age 75 to 84.^1^ Thus, more effective therapeutic strategies for treating GBM are urgently needed. Epigenetic studies have revealed that promoting methylation by silencing the *MGMT* gene is correlated to longer overall survival (OS) in GBM patients after receiving alkylating chemotherapy with carmustine or temozolomide during radiotherapy treatment.^4^, ^5^ The *MGMT* gene exists on chromosome 10q26 and encodes a protein that functions in DNA repair by removing alkyl groups from the O^6^ position of guanine, which plays a key role in DNA alkylation in the process of DNA repair.^6^ The protein of MGMT could be consumed by the restoration of the DNA content, and then the cell replenished after DNA repair could be restored in those processes.^7^ O^6^‐methylguanine, which is produced after DNA damage, induces cytotoxicity and apoptosis.^7^ Overexpression of MGMT protein in cancer cells could form a resistant phenotype by attenuating the therapeutic effect after treatment with alkylating agents in GBM and contribute to treatment failure.^8^, ^9^, ^10^ An epigenetic strategy was applied to silence the *MGMT* gene with promoter methylation, and silencing of the *MGMT* gene has been found to be related to the loss of MGMT protein expression, causing a reduction in DNA repair activity.^11^, ^12^, ^13^

Omics methods have been applied to explore the molecular changes and mechanisms in GBM.^14^, ^15^, ^16^, ^17^ Integrated metabolomics and proteomics analysis have been applied to explore global proteome and metabolome levels in GBM.^18^, ^19^, ^20^ Metabolomics and proteomics analyses are complementary to other omics, including genomics, epigenetics and transcriptomics, and directly reflect the physiological status of GBM.^21^ With the rapid development of liquid chromatography–mass spectrometry (LC–MS)‐based “omics” methods, metabolomics and proteomics analyses have been applied to analyze metabolite or protein level patterns in biological samples, providing valuable information for biomarker screening and pathological research.^22^

In this study, we explored the dysregulated proteins and metabolites of GBM patients with or without the *MGMT* gene by integrating proteomics and metabolomics. Our results provide novel mechanisms for understanding the methylation in the GBM and identify some biomarkers for prognosis of two different GBM types of MGMT promoter unmethylated or methylated GBM, and reveal the fundamental differences between those groups; this paper also emphasizes the available treatment strategies for GBM. Our proteomics and metabolomics results could provide a novel window into the role of MGMT in GMB during clinical practice.

## 2 METHODS

### 2.1 Human samples

Glioblastoma (GBM) samples were collected from glioblastoma patients undergoing surgery with procedures approved by the Ethics Committee of the First Affiliated Hospital of Xiamen University from October 2021 to December 2022. All subjects (*n* = 8 patients for each group) provided written informed consent in this study. For control group samples, all human white matter samples (*n* = 8) were obtained from the Chinese Brain Bank Center (CBBC).

The levels of MGMT promoter methylation were measured by Cheerland Biotechnology Co., Ltd. All enrolled samples received next‐generation gene sequencing (NGS) to measure the gene expression of *MGMT*.

In this study, three groups, including the control group (CON), glioblastoma group (GBM), and MGMT expression in the GBM group (MGMT), were used to perform proteomic and metabolomic analyses. All samples were immediately collected in liquid nitrogen and stored at −80°C until the following experiments were conducted.

### 2.2 Proteomics analysis

The samples of eight randomized white matter controls, eight randomized GBM groups, and eight randomized MGMT groups were mixed into three pooled samples for proteomics analysis. The proteomics analysis experiments were performed according to previous studies.^23^, ^24^ All the samples were homogenized by using SDT lysis buffer including 4% SDS, 100 mM dithiothreitol, 100 mM Tris–HCl, pH 8.0, and protease inhibitors. The samples were incubated at 100°C for 5 min and then centrifuged for 10 min at 40 000 *g*. The protein concentrations were measured by using a Pierce bicinchoninic acid assay.

The filter‐aided sample preparation (FASP)‐based protocol with 10 kDa ultrafiltration centrifuge tubes was applied for protein digestion. Briefly, the sample lysates were diluted by using a urea solution containing 150 mM Tris–HCl, pH 8.0, and 8 M urea. The proteins in the sample lysates were alkylated by using 50 mM iodoacetamide for 30 min in the dark. The alkylated proteins were washed twice by using the urea solution. All the protein samples were digested by using trypsin for more than 12 h at 37°C. The peptide products were collected by centrifugation and washing. Then, the peptides were dried in a Speed Vac.

The peptides were labeled with 8‐plex isobaric tags for relative and absolute quantitation (iTRAQ) reagents according to the manufacturer's instructions. All samples were subjected to liquid chromatography–tandem mass spectrometry (LC–MS/MS) analysis. Then, tandem mass spectrometry spectra were analyzed by using the MASCOT engine 2.2 (Matrix Science). The dysregulated proteins were recognized by using a standard including fold change values of greater than ±1.2 and *p* values of less than 0.05. Gene Ontology (GO) enrichment, including cellular component, molecular function, and biological process, and Kyoto Encyclopedia of Genes and Genomes (KEGG) pathway enrichment analyses were performed by using Fisher's exact test.

### 2.3 Metabolomics analysis

A Waters UPLC I‐class system equipped with a binary solvent delivery manager (Waters Corporation) was applied to perform untargeted liquid chromatography–mass spectrometry‐based metabolomics (eight samples per group), and the detailed protocols were performed according to a previous study with minor modifications.^25^ All samples stored at −80°C were thawed on ice for 10 min. 2‐Chloro‐1‐phenylalanine, which was dissolved in methanol (0.3 mg/mL), was applied to an internal standard. In an Eppendorf tube, 50 mg of sample and 10 μL of internal standard were mixed and vortexed. Then, 150 μL of an ice‐cold mixture of methanol and acetonitrile (2/L, vol/vol) was added into the Eppendorf tube. All the mixtures were vortexed for approximately 1 min, ultrasonicated at 25°C for 5 min, placed at −20°C for 10 min, and centrifuged at 40,000 *g* at 4°C for 10 min. The supernatants (100 μL) from each Eppendorf tube were collected and filtered by using 0.22 microfilters and then subjected to LC–MS analysis.

The LC–MS analysis data were collected by using a Waters VION IMS Q‐TOF Mass Spectrometer equipped with an electrospray ionization (ESI) source operating in either positive or negative ion mode. The data, including *m*/*z*, peak RT, and peak intensities, were analyzed by using the Human Metabolome Database (HMDB, http://www.hmdb.ca), Metlin (https://metlin.scripps.edu), and LipidMaps (http://www.lipidmaps.org). The positive and negative data were combined and imported into the SIMCA‐P+ 13.0 software package (Umetrics) for multivariate statistical analysis. An orthogonal partial least squares‐discriminant analysis (OPLS‐DA) model was applied to exert significant differences and identify differentially expressed metabolites in GBM patients with or without MGMT.

### 2.4 Integrated analysis

Ingenuity Pathway Analysis software (IPA, QIAGEN) was applied to explore metabolic pathways associated with the differentially expressed metabolites and proteins according to previous studies.^26^, ^27^ We uploaded the lists and fold change values of differentially expressed proteins or metabolites to IPA software. IPA software was applied to calculate a *p* score for each of the possible networks in accordance with the fit homology to all the input molecules. This score is derived from a *p* value and indicates the probability of the input molecules in a given network to coexist as a result of random chance [*p* score = −log_10_ (*p* value)].

### 2.5 Statistical analysis

We performed statistical analyses using GraphPad Prism 7.0 (GraphPad Software), and the data are presented as the mean ± standard error of the mean (SEM). The Shapiro–Wilk test for normality was applied to assess data distribution. Unpaired *t* tests were performed for metabolomics and proteomics analysis between two groups (CON vs. GBM and GBM vs. MGMT). Fold change (FC) ≥2 and *p* < 0.05 were considered to be significant differences during proteomics and metabolomics analysis. KEGG enrichment analysis was applied to explore the metabolic pathways. The results of gender analysis in human samples were subjected to *χ* ^2^ tests. The details of all statistical analyses are described in the figure legends.

## 3 RESULTS

### 3.1 Workflow of proteomic and metabolomic analysis

**FIGURE 1:**
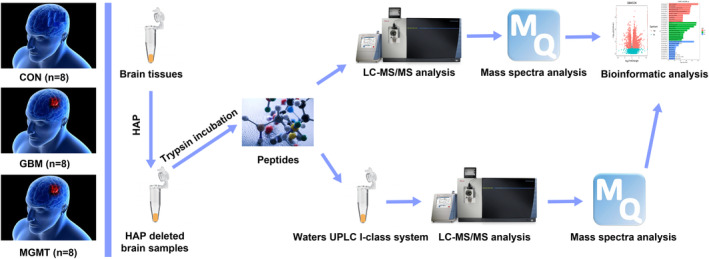
The workflow of proteomic and metabolomic analysis in this study. The highest abundance proteins (HAPs) in the brain tissues were removed by using Pierce™ TOP Abundant Protein Depletion Spin Columns according to the manufacturer's instructions. Then, the HAP‐deleted brain samples were digested by using trypsin‐based FASP. For proteomic experiments, all the digested peptides were labeled with iTRAQ reagents and subjected to a Q Exactive HFLC–MS/MS instrument (Thermo). Then, all the mass spectra were searched against the human UniProt database using MaxQuant software, and bioinformatics analysis was performed. For metabolomic analysis, a Waters UPLC I‐class system equipped with a binary solvent delivery manager was applied to perform untargeted liquid chromatography–mass spectrometry‐based metabolomics after digestion of peptides. All the raw data were analyzed by using MaxQuant software and processed by using MaxQuant software before OPLS‐DA. Ingenuity Pathway Analysis software was applied to explore metabolic pathways associated with the differentially expressed metabolites and proteins.

Proteomic and metabolomic analyses were performed using eight human white matter samples for the control group, eight glioblastoma (GBM) samples and eight MGMT‐positive GBM samples as shown in Table 1. The highest abundance proteins (HAPs) in the brain tissues were removed by using Pierce™ TOP Abundant Protein Depletion Spin Columns according to the manufacturer's instructions. Then, the HAP‐deleted brain samples were digested by using trypsin‐based FASP. For proteomic experiments, all the digested peptides were labeled with iTRAQ reagents and subjected to a Q Exactive HFLC–MS/MS instrument (Thermo). Then, all the mass spectra were searched against the human UniProt database using MaxQuant software, and bioinformatics analysis was performed. For metabolomic analysis, a Waters UPLC I‐class system equipped with a binary solvent delivery manager was applied to perform untargeted liquid chromatography–mass spectrometry‐based metabolomics after the digestion of peptides. All the raw data were analyzed by using MaxQuant software and processed by using MaxQuant software before OPLS‐DA. Ingenuity Pathway Analysis software was applied to explore metabolic pathways associated with the differentially expressed metabolites and proteins (Figure 1).

**TABLE 1:** The information of all enrolled patients and healthy controls in this study.

Variable (SEM/%)	CON	GBM	*p*‐Value	GBM	MGMT	*p*‐Value
Cases (*n*)	8	8	/	8	8	/
Age (years)	47.5 ± 1.3	44.2 ± 6.5	0.63	44.2 ± 6.5	48.8 ± 5.5	0.59
Gender						
Male (%)	37.5	37.5	1	37.5	37.5	1
Female (%)	62.5	62.5	1	62.5	62.5	1
MGMT (%)	0	0	1	0	51.0 ± 8.0	<0.0001

Abbreviations: CON, healthy controls; GBM, glioblastoma; MGMT, O^6^‐methylguanine‐DNA methyltransferase; SEM, standard error of the mean.

### 3.2 Proteomics profiling analysis of white matter and GBM human samples

**FIGURE 2:**
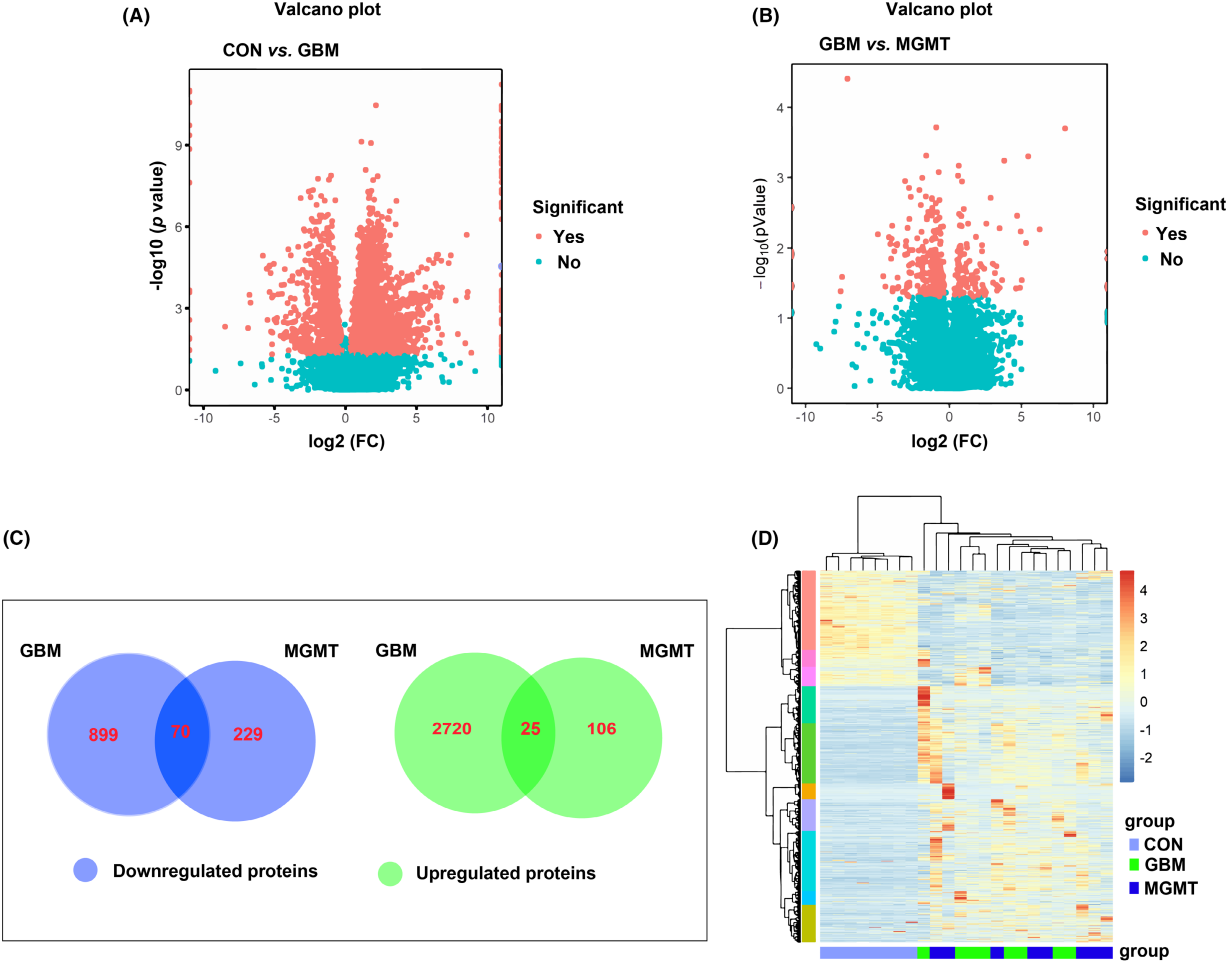
Analysis of the differentially regulated proteins from the control group (CON), glioblastoma group (GBM) and MGMT expression in GBM group (MGMT). The volcano plots of CON vs. GBM (A) or GBM vs. MGMT (B) showed the differentially expressed proteins between those two groups. (C) Venn diagrams illustrating the number of downregulated and upregulated proteins between CON vs. GBM or GBM vs. MGMT. (D) Heatmap‐based clustering of differentially regulated proteins identified in those three groups. The intensities of various colors illustrate the expression levels. The color bar is log_2_ scaled.

Quantitative proteomics analysis was conducted in the control group, GBM group, and MGMT group by using an iTRAQ‐based quantitative strategy. Eight samples for each group in white matter or GBM tissues were analyzed in this study. Then, all the proteomic results were subjected to independent hypotheses and were not adjusted for multiple comparisons. The results of *p* values less than 0.05 and fold change (FC) greater than 2 were regarded to be suggestive of trends in this study. A total of 8717 nonredundant proteins were identified and analyzed with a false discovery rate (FDR) of less than 1% (Table S1). Volcano plots showed that a total of 2745 proteins were upregulated and 969 proteins were downregulated in the GBM group compared to the control group (Figure 2A), and 131 proteins were upregulated and 299 proteins were downregulated in the MGMT group compared to the GBM group (Figure 2B). The results of proteomics profiling analysis showed that 3714 proteins were dysregulated in the GBM group compared to the control group. Among those proteins, we found 70 proteins with similar downregulation between the GBM group and the MGMT group (Figure 2C and Table S2). Moreover, we found 25 proteins with similar upregulation between the GBM group and the MGMT group (Figure 2C and Table S3). Further analysis of heatmap‐based clustering of 3714 dysregulated proteins in those three groups reflected the possible responses in GBM with or without the *MGMT* gene (Figure 2D).

**FIGURE 3:**
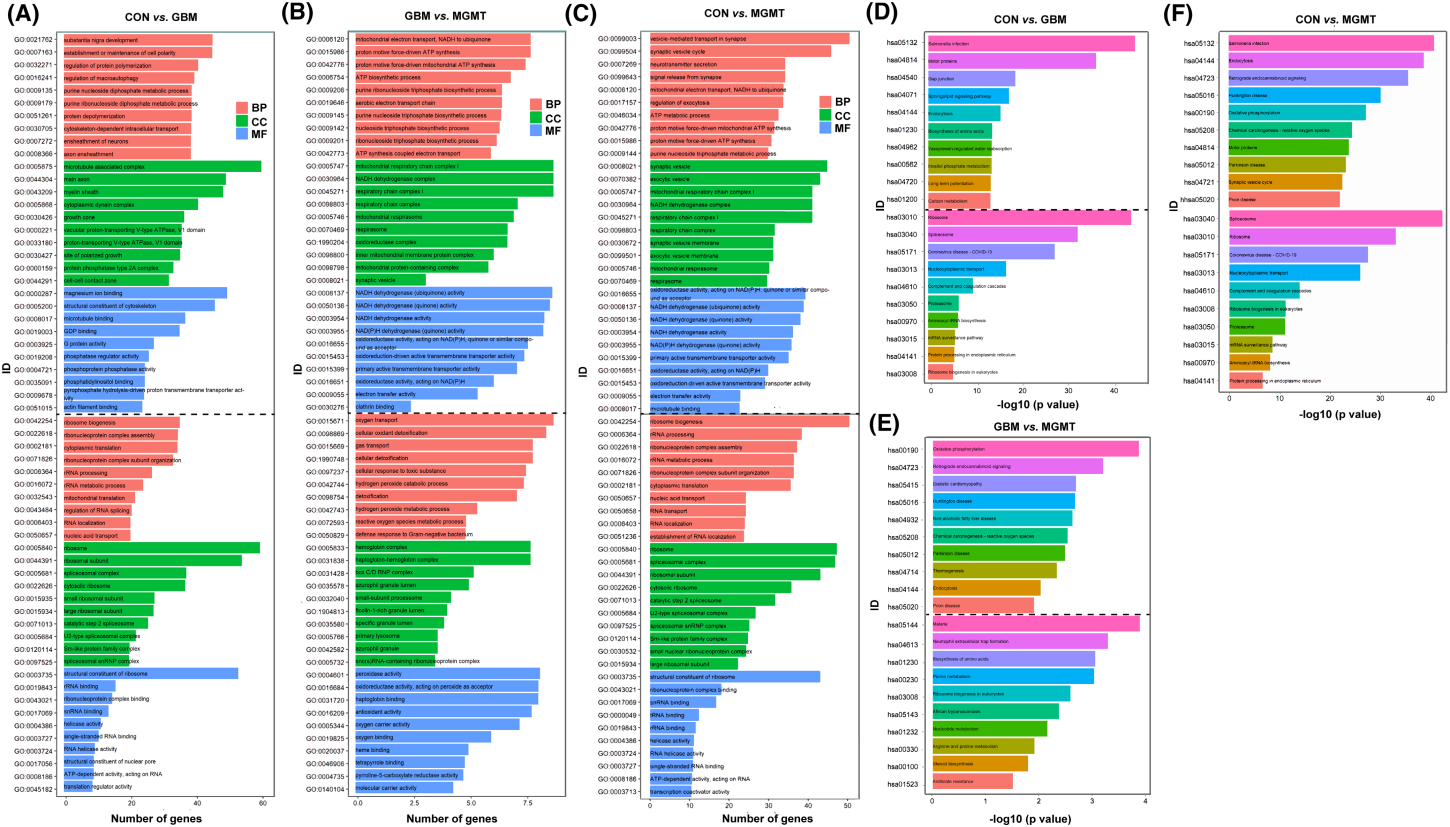
Gene Ontology (GO) and Kyoto Encyclopedia of Genes and Genomes (KEGG) pathway enrichment analysis of the differentially regulated proteins from the control group (CON), glioblastoma group (GBM) and MGMT expression in GBM group (MGMT). (A) The top 20 enriched GO biological process (BP), cellular component (CC), and molecular function (MF) terms are illustrated in CON vs. GBM. (B) The top 20 enriched BP, CC and MF terms are illustrated in GBM vs. MGMT. (C) The top 20 enriched BP, CC and MF terms are illustrated in CON vs. MGMT. (D) The significantly enriched KEGG pathway terms in CON vs. GBM. (E) The significantly enriched KEGG pathway terms in GBM vs. MGMT. (F) The significantly enriched KEGG pathway terms in CON vs. MGMT. The X‐axis represents the log_10_ negative *p* value.

To explore the significant biological functions and signaling pathways related to the *MGMT* gene in GBM, gene ontology (GO) enrichment, including cellular component, molecular function, and biological process, and Kyoto Encyclopedia of Genes and Genomes (KEGG) pathway enrichment analyses were performed in this study. The dysregulated proteins in the CON vs. GBM, GBM vs. MGMT and CON vs. MGMT groups were subjected to bioinformatics analysis. A total of 4307, 3863 and 1992 terms in the biological process (BP), cellular component (CC), molecular function (MF) and KEGG pathways were considered to be significantly overrepresented, respectively. In this study, 20 enriched GO biological processes were listed, including 10 downregulated proteins (upper panel) and 10 upregulated proteins (lower panel) (Figure 3). BP analysis showed that many proteins were involved in oxygen transport, cellular oxidant detoxification, ribosome biogenesis and ribonucleoprotein complex assembly (Figure 3A–C, and Table S4). A large number of proteins in the CC category were related to ribosomes, hemoglobin complexes and mitochondrial respirasomes (Figure 3A–C, and Table S5). Moreover, most proteins in the MF category were mainly related to structural constituent of ribosome, peroxidase activity, oxidoreductase activity, haptoglobin binding, antiboidant activity and oxygen carrier activity (Figure 3A–C, and Table S6). The enrichment of KEGG pathways illustrated that those dysregulated proteins were mainly related to Salmonella infection, spliceosome, oxidative phosphorylation and Malaria (Figure 3D–F, and Table S7).

Meanwhile, GO and KEGG pathway enrichment analyses of 2745 upregulated proteins and 969 downregulated proteins in the GBM vs. CON group were performed in this study. A total of 4307 BP, 3863 CC, 1992 MF and 204 KEGG pathway terms were significantly enriched in the CON vs. GBM group. The top 20 enriched BP, CC, MF (Figure 3A) and 615 KEGG pathway terms (Figure 3E). The analysis of BP classification showed that most proteins were involved in substantia nigra development, establishment or maintenance of ribosome biogenesis, ribonucleoprotein complex assembly, cytoplasmic translation, cell polarity and regulation of protein polymerization. The analysis of CC classification showed that most proteins were related to ribosomes, ribosomal subunits, spliceosomal complexes, microtubule‐associated complexes, main axons and myelin sheaths. Meanwhile, the analysis of MF revealed that most proteins were related to structural constituents of ribosomes, rRNA binding, ribonucleoprotein complex binding, magnesium ion binding, structural constituents of the cytoskeleton and microtubule binding (Figure 3A). KEGG pathway analysis revealed that the dysregulated proteins were mainly related to Salmonella infection, motor proteins and ribosomes (Figure 3D).

Furthermore, GO and KEGG pathway enrichment analyses of 131 upregulated proteins and 299 downregulated proteins in the GBM vs. MGMT group were performed in this study. Our results showed that 392 BP, 683 CC, 1019 MF and 448 KEGG pathway terms were significantly enriched in the GBM vs. MGMT group. The analysis of BP classification showed that most proteins were involved in oxygen transport, cellular oxidant detoxification, gas transport, mitochondrial electron transport, proton motive force‐driven ATP synthesis and ATP biosynthetic processes. The analysis of CC classification showed that most proteins were related to the hemoglobin complex, haptoglobin‐hemoglobin complex, Box C/D RNP complex, mitochondrial respiratory chain complex I, NADH dehydrogenase complex and respiratory chain complex I. Meanwhile, the analysis of MF revealed that most proteins were related to peroxidase activity, oxidoreductase activity, haptoglobin binding, NADH dehydrogenase (ubiquinone) activity, NADH dehydrogenase (quinone) activity and NADH dehydrogenase activity (Figure 3B). KEGG pathway analysis revealed that the dysregulated proteins were mainly related to oxidative phosphorylation, retrograde endocannabinoid signaling, malaria, neutrophil extracellular trap formation and biosynthesis of amino acids (Figure 3E).

**FIGURE 4:**
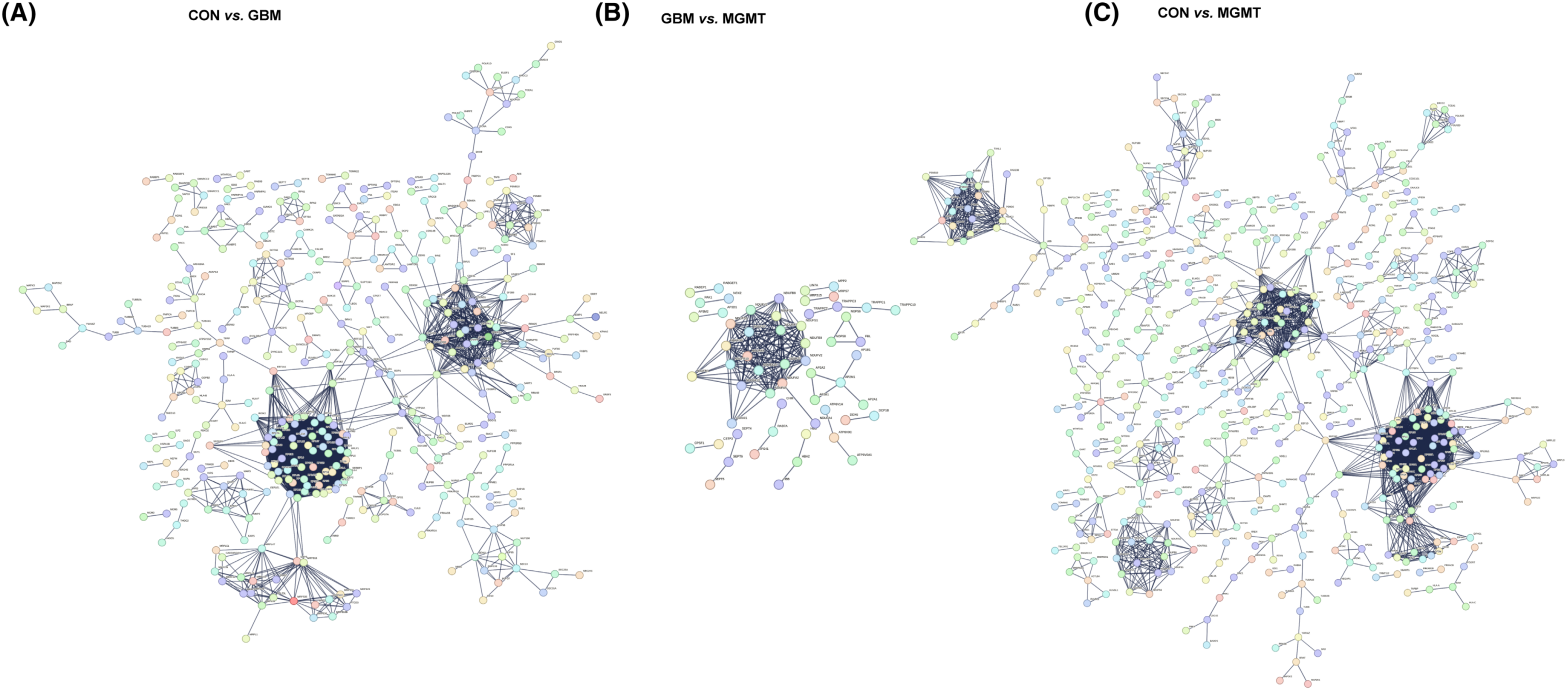
Protein–protein interaction (PPI) analysis of the differentially regulated proteins in the control group (CON), glioblastoma group (GBM) and MGMT expression in GBM group (MGMT). (A) The PPI networks of CON vs. GBM were built on the basis of altered protein expression and overrepresented Kyoto Encyclopedia of Genes and Genomes (KEGG) pathways. (B) The PPI networks of GBM vs. MGMT were built in this study. (C) The PPI networks of CON vs. MGMT were built in this study. Proteins/genes are indicated with circular nodes.

Moreover, protein–protein interaction (PPI) networks were built based on the proteomics results in the CON vs. GBM group (Figure 4A), GBM vs. MGMT group (Figure 4B) or CON vs. MGMT group (Figure 4C). The PPI networks of those three group pairs were created by using the significantly enriched KEGG pathways that were built by using dysregulated proteins. Based on a unified conceptual framework, we identified 304, 39 and 615 proteins as significant nodes in the PPI networks from the CON vs. GBM group, GBM vs. MGMT group, and CON vs. MGMT group, respectively. The PPI networks revealed the KEGG pathways and the corresponding dysregulated proteins and their close correlations and then provided a small pool of interactomes that illustrated the potential mechanisms of MGMT‐positive GBM.

### 3.3 Metabolomic analysis of white matter and GBM human samples

**FIGURE 5:**
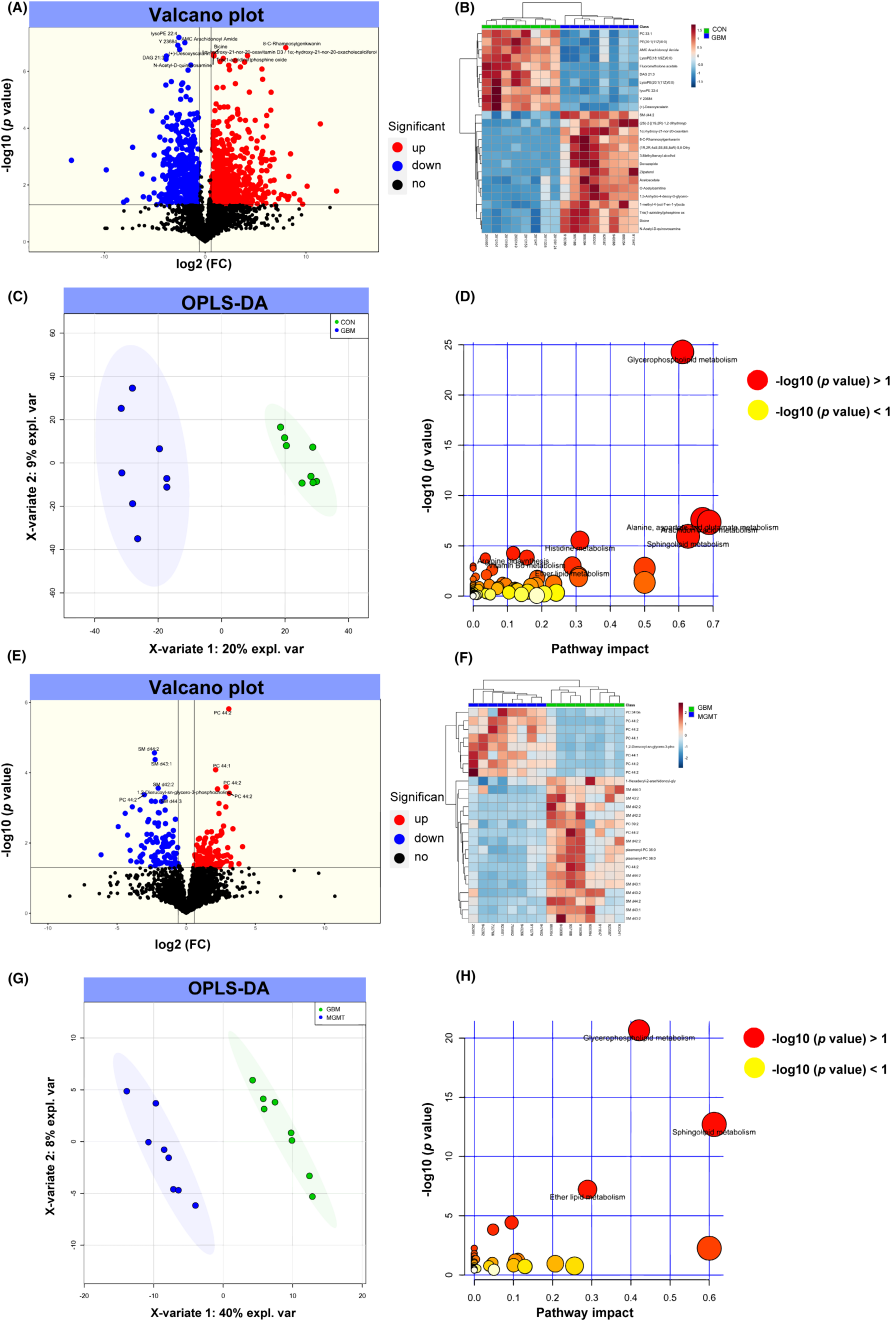
Metabolomic profiling analysis of differentially regulated metabolites from the control group (CON), glioblastoma group (GBM) and MGMT expression in GBM group (MGMT). The volcano plot (A) and heatmap (B) show the differentially expressed metabolites in CON vs. GBM. (C) An orthogonal partial least squares‐discriminant analysis (OPLS‐DA) showed the differentially expressed metabolites in CON vs. GBM. (D) KEGG analysis of differentially expressed metabolites in CON vs. GBM. The volcano plot (E) and heatmap (F) show the differentially expressed metabolites in GBM vs. MGMT. (G) OPLS‐DA showed the differentially expressed metabolites in GBM vs. MGMT. (H) KEGG analysis of differentially expressed metabolites in GBM vs. MGMT.

We performed metabolomic analysis in the control group, GBM group and MGMT group. The differential analysis showed 864 upregulated and 665 downregulated metabolites in the GBM group compared to the CON group (Figure 5A). Further analysis of heatmap‐based clustering of 1529 dysregulated metabolites in those two groups reflected the possible responses in GBM compared to the CON group (Figure 5B). Notably, the levels of Cynarasaponin J and Fisetinidol were the most significant, with FCs of 0.00010151 and 8599.5, respectively. The orthogonal partial least squares‐discriminant analysis (OPLS‐DA) results of the CON vs. GBM groups are shown in Figure 5C. The dysregulated metabolites in the GBM group compared to the CON group were mainly enriched in glycerophospholipid metabolism, alanine, aspartate and glutamate metabolism and arachidonic acid metabolism (Figure 5D, and Table S8). The differential analysis showed 187 upregulated and 147 downregulated metabolites in the MGMT group compared to the GBM group (Figure 5E). Further heatmap‐based clustering analysis of 334 dysregulated metabolites in those two groups reflected the possible responses in the MGMT group compared to the GBM group (Figure 5F). Moreover, OPLS‐DA showed that the metabolomics maps of the GBM vs. MGMT group changed greatly compared to those of the CON vs. GBM group (Figure 5C,G). The dysregulated metabolites in the MGMT group compared to the GBM group were mainly enriched in glycerophospholipid metabolism, sphingolipid metabolism and tyrosine metabolism (Figure 5H, and Table S9).

### 3.4 Integrated analysis of differentially expressed proteins and metabolites

**FIGURE 6:**
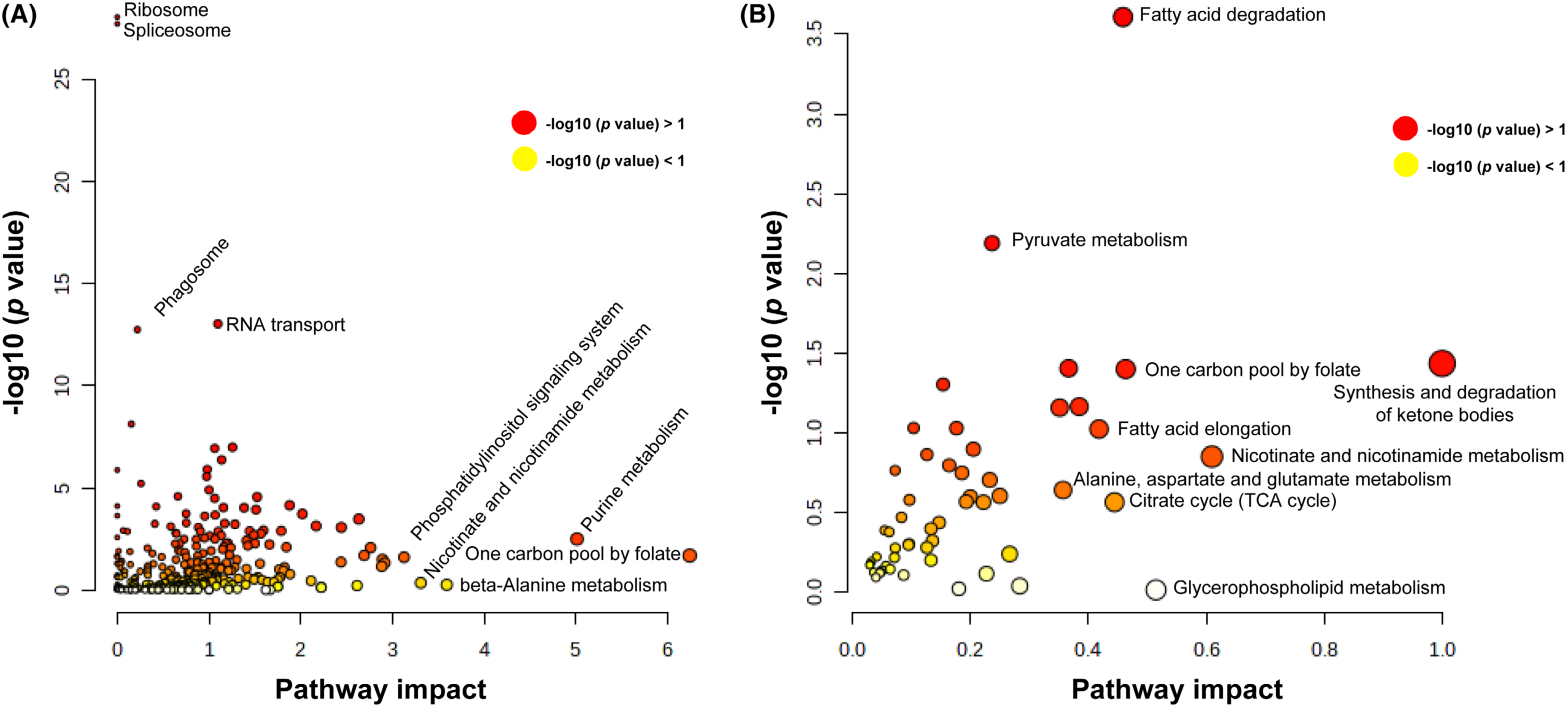
Integrated analysis of metabolomics and proteomics analyses of the control group (CON), glioblastoma group (GBM) and MGMT expression in GBM group (MGMT). (A) Integrated analysis of metabolomics and proteomics analyses in CON vs. GBM. (B) Integrated analysis of metabolomics and proteomics analyses in GBM vs. MGMT.

In this study, we screened out the metabolites and proteins with FC >2 or FC <0.5 in the CON, GBM and MGMT groups in human samples. In this study, 571 metabolites and 3714 proteins were found in the CON vs. GBM group pairs and were subjected to Ingenuity Pathway Analysis (IPA) software for integrated analysis of differentially expressed proteins and metabolites. The results showed that the one‐carbon pool by folate, ribosomes and spliceosomes played key roles in GBM compared to the CON group (Figure 6A, and Table S10). Moreover, 150 metabolites and 430 proteins were found in the GBM vs. MGMT group pairs and subjected to IPA software for integrated analysis of differentially expressed proteins and metabolites. The results showed that the synthesis and degradation of ketone bodies, glycerophospholipid metabolism and fatty acid degradation played key roles in the MGMT group compared to the GBM group. Moreover, the results showed that 94 upregulated and 19 downregulated proteins and 20 upregulated and 16 downregulated metabolites in the MGMT group were associated with DNA repair (Figure 6B, and Table S11). Moreover, integrated metabolomics and proteomics analysis was performed, and six key proteins, DENN domain containing 3, Ras protein‐specific guanine nucleotide releasing Factor 2 (RasGRF2), potassium voltage‐gated channel subfamily Q member 2, sprouty RTK signaling antagonist 2, unc‐5 netrin receptor C and glutathione S‐transferase alpha 1, were identified in the MGMT group and GBM group. Then, three key pathways, including the synthesis and degradation of ketone bodies, glycerophospholipid metabolism and fatty acid degradation, were recognized as potential biomarkers for recognizing MGMT promoter unmethylated GBM and MGMT promoter methylation positive GBM from GBM patient samples, with areas under the curve of 0.7895, 0.7326 and 0.7026, respectively.

## 4 DISCUSSION

Glioblastoma (GBM) is the most common and aggressive malignant brain tumor in clinical practice.^28^ Chemotherapy, surgical resection and radiation have been applied to treat GBM in clinical practice. The pharmacological treatment of GBM in clinical practice remains difficult due to the microenvironment of GBM and the blood–brain barrier (BBB).^29^ Temozolomide, a first‐line drug for GBM treatment, has been reported to increase resistance to marketed drugs during the treatment of GBM.^30^ The MGMT promoter methylation has been reported to be related to overall survival (OS) in GBM patients after temozolomide treatment in clinical practice.^31^, ^32^ In this study, we explored the dysregulated proteins and metabolites of GBM patients with or without the *MGMT* gene by integrating proteomics and metabolomics. Our results provide novel mechanisms for understanding the methylation in the GBM and identify some biomarkers for prognosis of two different GBM types of MGMT promoter unmethylated or methylated GBM; we also reveal the fundamental differences between those groups and emphasize the available treatment strategies for GBM. Our proteomics and metabolomics results provide a novel window into understanding the role of MGMT in GMB during clinical practice.

Proteomics and metabolomics were applied to explore potential mechanisms at large‐scale levels and then study the pathological progression of diseases.^33^, ^34^ Moreover, proteomics and metabolomics can more deeply reflect disease progression than genetic omics.^35^ Recently, a number of studies have performed proteomics and metabolomics to explore the potential mechanisms of GBM.^14^, ^16^, ^36^, ^37^ For example, Ravi et al.^37^ characterized glioblastomas by spatially resolved transcriptomics, metabolomics, and proteomics. Masui et al.^36^ reported that the metabolomic landscape plays a critical role in glioma oncogenesis. Semer Maksoud has reviewed that the DNA double‐strand breaks and their repair in the progression of gliomas.^38^ However, the proteomic and metabolomic patterns in MGMT‐positive GBM remain largely unknown. In this study, proteomics and metabolomics were combined to explore the dysregulated metabolites and possible protein expression alterations in white matter (control group), MGMT promoter unmethylated GBM (GBM group) or MGMT promoter methylation positive GBM (MGMT group). The results showed that 2745 proteins were upregulated and 969 proteins were downregulated in the GBM group compared to the control group, and 131 proteins were upregulated and 299 proteins were downregulated in the MGMT group compared to the GBM group. Meanwhile, 864 upregulated and 665 downregulated metabolites were identified in the GBM group compared to the CON group, and 187 upregulated and 147 downregulated metabolites were identified in the MGMT group compared to the GBM group. The dysregulated metabolites in the MGMT group compared to the GBM group were mainly enriched in glycerophospholipid metabolism, sphingolipid metabolism and tyrosine metabolism. The analysis of KEGG pathways revealed that the dysregulated proteins were mainly related to oxidative phosphorylation, retrograde endocannabinoid signaling, malaria, neutrophil extracellular trap formation and biosynthesis of amino acids in the MGMT group vs. GBM group. Furthermore, dysregulated metabolites in the MGMT group compared to the GBM group were mainly enriched in glycerophospholipid metabolism, sphingolipid metabolism and tyrosine metabolism. Moreover, the results showed that 94 upregulated and 19 downregulated proteins and 20 upregulated and 16 downregulated metabolites in the MGMT group were associated with DNA repair. Thus, we suspected that MGMT‐related DNA repair may become a potential target to treat GBM in clinical practice. The PPI networks revealed the KEGG pathways and the corresponding dysregulated proteins and their close correlations and then provided a small pool of interactomes that illustrated the potential mechanisms of MGMT‐positive GBM.

Integrated metabolomics and proteomics analysis was performed, and six key proteins, DENN domain containing 3, Ras protein‐specific guanine nucleotide releasing Factor 2 (RasGRF2), potassium voltage‐gated channel subfamily Q member 2, sprouty RTK signaling antagonist 2, unc‐5 netrin receptor C and glutathione S‐transferase alpha 1, were identified in the MGMT group and GBM group. Moreover, Shan et al.,^39^ have pointed that RasGRF2 has good stability and potential application value for poor prognosis in patients with glioma. Then, three key pathways, including the synthesis and degradation of ketone bodies, glycerophospholipid metabolism and fatty acid degradation, were recognized as potential biomarkers for recognizing MGMT promoter unmethylated GBM and MGMT promoter methylation positive GBM from GBM patient samples, with areas under the curve of 0.7895, 0.7326 and 0.7026, respectively. Cho et al.^40^ noted that RASGRF2 is highly related to progression in GBM. Ketone bodies for energy have been reported to be involved in tumor metabolism in GBM.^41^, ^42^ Thus, targeting the synthesis and degradation of ketone bodies may be a potential therapy for MGMT‐positive GBM. However, the detailed mechanisms of synthesis and degradation of ketone bodies in MGMT‐positive GBM remain unclear. These dysregulated proteins and metabolites can be used as potential clinical molecular markers for distinguishing two types of *MGMT* gene expression in GBM.

## 5 CONCLUSION

Through proteomic and metabolomic analyses, we screened dysregulated proteins and metabolites in the GBM vs. CON or MGMT vs. GBM groups. In total, 2745 upregulated and 969 downregulated proteins were identified in the GBM group compared to the control group, and 131 upregulated and 299 downregulated proteins were identified in the MGMT group compared to the GBM group. Furthermore, 131 upregulated and 299 downregulated metabolites were identified in the GBM group compared to the control group, and 187 upregulated and 147 downregulated metabolites were identified in the MGMT group compared to the GBM group. The results showed that 94 upregulated and 19 downregulated proteins and 20 upregulated and 16 downregulated metabolites in the MGMT group were associated with DNA repair. KEGG pathway enrichment analysis illustrated that the dysregulated proteins and metabolites were involved in multiple metabolic pathways, including the synthesis and degradation of ketone bodies, amino sugar and nucleotide sugar metabolism and starch and sucrose metabolism. Moreover, integrated metabolomics and proteomics analysis was performed, and six key proteins were identified in the MGMT group and GBM group. Then, three key pathways were recognized as potential biomarkers for recognizing MGMT promoter unmethylated GBM and MGMT promoter methylation positive GBM from GBM patient samples, with areas under the curve of 0.7895, 0.7326 and 0.7026, respectively. Thus, we suspected that those metabolites and proteins could be applied for molecular markers to identify those two GBM types in clinical practice. Overall, this study provides novel mechanisms for understanding methylation in GBM and identifies some biomarkers for the prognosis of two different GBM types, MGMT promoter unmethylated or methylated GBM, by using metabolomics and proteomics analyses. In summary, this study provided novel insight into the mechanisms underlying the development of MGMT‐positive GBM and identified novel biomarkers for the development of MGMT‐positive GBM and MGMT‐negative GBM by using metabolomics and proteomics analyses.

In this study, a limited progress has been made on the mechanisms and biomarkers in the MGMT‐positive GBM and MGMT‐negative GBM by using metabolomics and proteomics analyses. In our future studies, large sample sizes should be employed in GBM patients to explore its potential mechanisms.

## AUTHOR CONTRIBUTIONS

Xi Chen and Guowei Tan designed this study. Xi Chen, Jinli Sun, Yukui Li, Weichao Jiang, Zhangyu Li and Jianyao Mao assessed the clinical data. Xi Chen, Jinli Sun, Yukui Li, Liwei Zhou and Sifang Chen performed the experiments. Xi Chen, Jinli Sun, Yukui Li and Guowei Tan wrote and revised the manuscript. All authors approved the final version of the manuscript for publication.

## FUNDING INFORMATION

This study was funded by the Natural Science Foundation of Xiamen City, China (3502Z20227093), Medical and Health Guidance Program of Xiamen City, China (3502Z20214ZD1021 & 3502Z20224ZD1012), and Natural Science Foundation of Fujian Province, China (2021J011350).

## CONFLICT OF INTEREST STATEMENT

The authors declare no conflicts of interest.

## CONSENT TO PARTICIPATE

The studies involving human participants were reviewed and approved by the Ethics Committee of Scientific Research, The First Affiliated Hospital of Xiamen University. The patients/participants provided their written informed consent to participate in this study.

## Supporting information


Table S1.

## Data Availability

The data that support the findings of this study are available on request from the corresponding author. The data are not publicly available due to privacy or ethical restrictions.
