# Supplementary material for: Proteomic and metabolomic analyses illustrate the mechanisms of expression of the O6‐methylguanine‐DNA methyltransferase gene in glioblastoma
Source: CNS Neurosci Ther. 2023 Aug 28;30(2):e14415. doi: 10.1111/cns.14415 (PMC10848106; doi:10.1111/cns.14415)
Supplement: Supplementary file 1 — Table S1. [file CNS-30-e14415-s001.zip › Supplement tables 1-11 captions.doc]

**SUPPLEMENTARY MATERIAL**

**Supplementary Table S1** Identification of the dysregulated proteins in the three groups.

**Supplementary Table S2** Identification of down-regulated proteins in the MGMT group compared to GBM group.

**Supplementary Table S3** Identification of up-regulated proteins in the MGMT group compared to GBM group.

**Supplementary Table S4** Enrichment results of biological process (BP) terms of the differentially regulated proteins from the MGMT group *vs.* GBM group.

**Supplementary Table S5** Enrichment results of cellular component (CC) terms of the differentially regulated proteins from the MGMT group *vs.* GBM group.

**Supplementary Table S6** Enrichment results of molecular function (MF) terms of the differentially regulated proteins from the MGMT group *vs.* GBM group.

**Supplementary Table S7** Enrichment of KEGG pathways in the differentially regulated proteins from the MGMT group *vs.* GBM group.

**Supplementary Table S8** Identification of the dysregulated metabolites in the CON group *vs.* GBM group.

**Supplementary Table S9** Identification of the dysregulated metabolites in the MGMT group *vs.* GBM group.

**Supplementary Table S10** Identification of the dysregulated metabolites in the MGMT group *vs.* GBM group.
